# Down-weighting overlapping genes improves gene set analysis

**DOI:** 10.1186/1471-2105-13-136

**Published:** 2012-06-19

**Authors:** Adi Laurentiu Tarca, Sorin Draghici, Gaurav Bhatti, Roberto Romero

**Affiliations:** 1 Perinatology Research Branch, NICHD/NIH/DHHS, , Bethesda, Maryland, and Detroit, MI, USA; 2 Department of Computer Science, Wayne State University, Detroit, MI, USA; 3 Center for Molecular Medicine and Genetics, Wayne State University, Detroit, MI, USA; 4 Department of Clinical and Translational Science, Wayne State University, Detroit, MI, USA

**Keywords:** Gene expression, Gene set analysis, Pathway analysis, Overlapping gene sets

## Abstract

**Background:**

The identification of gene sets that are significantly impacted in a given condition based on microarray data is a crucial step in current life science research. Most gene set analysis methods treat genes equally, regardless how specific they are to a given gene set.

**Results:**

In this work we propose a new gene set analysis method that computes a gene set score as the mean of absolute values of weighted moderated gene t-scores. The gene weights are designed to emphasize the genes appearing in few gene sets, versus genes that appear in many gene sets. We demonstrate the usefulness of the method when analyzing gene sets that correspond to the KEGG pathways, and hence we called our method ***P**athway **A**nalysis with **D**own-weighting of **O**verlapping **G**enes* (**PADOG**). Unlike most gene set analysis methods which are validated through the analysis of 2-3 data sets followed by a human interpretation of the results, the validation employed here uses 24 different data sets and a completely objective assessment scheme that makes minimal assumptions and eliminates the need for possibly biased human assessments of the analysis results.

**Conclusions:**

PADOG significantly improves gene set ranking and boosts sensitivity of analysis using information already available in the gene expression profiles and the collection of gene sets to be analyzed. The advantages of PADOG over other existing approaches are shown to be stable to changes in the database of gene sets to be analyzed. PADOG was implemented as an R package available at: http://bioinformaticsprb.med.wayne.edu/PADOG/or http://www.bioconductor.org.

## Background

Microarray-based gene expression profiling experiments, which are routine today, allow researchers to identify, for instance, genes differentially expressed (DE) between diseased and normal patient samples or genes that change in expression over time during a treatment. Unfortunately, the steady increase in the amount of data generated in the past decade from such experiments was not paralleled by the evolution of analytical methods used to extract knowledge from such datasets and, therefore, there is a gap between our ability to measure gene expression data and to extract workable knowledge from it.

Since the beginning of the microarray-based expression profiling experiments, researchers were interested in finding common “themes” among the genes identified as differentially expressed between two conditions. For instance the identification of Gene Ontology (GO) terms enriched in differentially expressed genes was used as early as 1999 [1], but became widespread only after the first on-line GO analysis tools were made available [2,3]. As biological annotations started to include descriptions of gene interactions in the form of pathways (found in resources such as KEGG [4], BioCarta http://www.biocarta.com, and Reactome [5]), the identification of the pathways involved in various conditions has emerged as a ubiquitous bioinformatics task.

In general, biological pathways can be divided into gene signaling pathways, and metabolic pathways. Gene signaling pathways are graphs that use nodes to represent genes, or gene products, and edges to represent signals that go from one gene to another. Metabolic pathways are graphs that use nodes to represent biochemical compounds, and edges to describe biochemical reactions that involve such compounds. Since biochemical reactions are usually carried out by enzymes which are coded for by genes, in a metabolic pathway genes are associated with edges rather than nodes. Ideally, a comprehensive pathway analysis method would be able to take into consideration all aspects of the phenomena described by a pathway. These aspects would include the position and role of each gene in a pathway, the types of signals between genes, the efficiency with which a signal travels from one gene to another, or the efficiency with which a certain reaction is carried out, rate limiting conditions, etc. Such methods have been proposed for both signaling pathways [6-9], and metabolic pathways [8,10], but no method is currently available to analyze both types of pathways taking into consideration all the information available. Hence, even though they do not use all information available, methods that treat the pathways as simple gene sets are still popular because they can be applied equally well to signaling pathways, metabolic pathways, GO terms, as well as arbitrary sets of genes.

Two of the most popular such methods are the *Gene Set Enrichment Analysis* (GSEA) [11] and the *Gene Set Analysis* (GSA) [12]. These methods belong to the *functional class scoring* category of gene set analysis methods [13,14]. For a simple two group experiment (e.g. disease vs. normal), both GSEA and GSA start with computing a t-statistic for each gene measured on the array. Then, a score is computed for each gene set using the *t*-scores of all genes in the gene set. The significance of the gene set scores is determined by using permutations of the samples. Both approaches treat the genes in the gene set equally.

In this work, we propose the ***P**athway **A**nalysis with **D**own-weighting of **O**verlapping **G**enes* (**PADOG**) which is a general gene set analysis method. The method gives more weight to genes that are gene set-specific, than to genes which can be found in multiple gene sets. This is similar to the approach commonly used in information retrieval (e.g. web search engines) that decreases the importance of words that appear in many documents (e.g. “and”, “or”, etc.) in favor of words that are highly specific to given documents, the latter type being considered to carry more information about the informational content of the document. Similarly, in our approach, if the differential expression affects genes that are highly specific to a given pathway (e.g. huntingtin to Hungtington’s disease), it is more likely that the respective pathway is truly relevant in that condition.

The process of down-weighting popular genes does not affect one’s ability to find a gene set to be significant whenever the gene set is composed mostly of ubiquitous genes, but rather increase the contrast between gene sets that overlap by reducing the contribution of the overlapping genes into the gene set scores. As a simple example, with PADOG, a gene set A having 20 out of 50 genes differentially expressed, that appear only in gene set A, will be found more significant than another gene set B of same size that has also 20 differentially expressed genes but which appear in other gene sets as well. Both GSEA and GSA would find the two gene sets equally significant.

Analysis methods that do not treat all genes equally were previously proposed for pathway analysis in an over-representation context [6,7], or in a functional class scoring context [8], yet none specifically exploit the frequency of occurrence of genes across the pathways. Moreover, unlike GSA, PADOG does not rely on ordinary *t*-scores to derive gene set scores but uses moderated t-statistics [15] instead. A similar idea to use non-ordinary *t*-scores in the gene set scores computation was illustrated first in [16] by using SAM statistics [17] in conjunction with GSEA. Moreover, unlike GSA, PADOG summarizes the gene scores into a gene set score using the mean of absolute values instead of the maxmean statistic.

The sensitivity of gene set analysis methods (i.e. their ability to produce significant *p*-values for gene sets that are truly relevant to a phenotype), as well their ability to rank the relevant gene sets near the top, is typically assessed using a few data sets, by asking domain experts to make informed guesses about which gene sets are relevant to each condition/dataset. Relevance is determined using the expert’s knowledge and/or literature citations supporting the link between certain gene sets and the condition under the study [6,7,11,18]. The problem is that almost any gene set analysis result will be supported by *some* references which makes an unbiased and objective comparison of various analysis methods practically impossible. In this study, we used a different approach in which we make fewer assumptions, and use an order of magnitude more data sets (24 sets). The type of gene sets considered in our validation were KEGG biological pathways. Each of the 24 microarray data sets that we used (see Table 1) involved a particular disease for which there is an associated pathway in the KEGG database [19], e.g. *Alzheimer’s diseaseColorectal cancerAsthma*, etc. We refer to these as the *target* pathways, and we, very conservatively, consider them to be the only ones certain to be relevant for their respective conditions. Since the target pathways for all 24 datasets belong to the non-metabolic pathways category, we can restrict the analysis only to KEGG non-metabolic pathways. Analyzing all metabolic and non-metabolic pathways brings an additional challenge to the analysis methods because the assumed relevant pathway for a given condition (dataset) is now to be found among a larger pool of pathways. The gene set analysis methods were compared in terms of their ability to produce significant *p*-values for these target pathways and rank them near the top.

**Table 1:** The 24 data sets used to assess the proposed gene set analysis method

	**GEOID**	**Pubmed**	**Ref.**	**Disease/Target pathway**	**KEGGID**	**Tissue**
1	GSE1297	14769913	[20]	Alzheimer’s Disease	hsa05010	Hippocampal CA1
2	GSE5281	17077275	[21]	Alzheimer’s Disease	hsa05010	Brain, Entorhinal Cortex
3	GSE5281	17077275	[21]	Alzheimer’s Disease	hsa05010	Brain, hippocampus
4	GSE5281	17077275	[21]	Alzheimer’s Disease	hsa05010	Brain, Primary visual cortex
5	GSE20153	20926834	[22]	Parkinson’s disease	hsa05012	Lymphoblasts
6	GSE20291	15965975	[23]	Parkinson’s disease	hsa05012	Postmortem brain putamen
7	GSE8762	17724341	[24]	Huntington’s disease	hsa05016	Lymphocytes (blood)
8	GSE4107	17317818	[25]	Colorectal Cancer	hsa05210	Mucosa
9	GSE8671	18171984	[26]	Colorectal Cancer	hsa05210	Colon
10	GSE9348	20143136	[27]	Colorectal Cancer	hsa05210	Colon
11	GSE14762	19252501	[28]	Renal Cancer	hsa05211	Kidney
12	GSE781	14641932	[29]	Renal Cancer	hsa05211	Kidney
13	GSE15471	19260470	[30]	Pancreatic Cancer	hsa05212	Pancreas
14	GSE16515	19732725	[31]	Pancreatic Cancer	hsa05212	Pancreas
15	GSE19728		-	Glioma	hsa05214	Brain
16	GSE21354		-	Glioma	hsa05214	Brain, Spine
17	GSE6956	18245496	[32]	Prostate Cancer	hsa05215	Prostate
18	GSE6956	18245496	[32]	Prostate Cancer	hsa05215	Prostate
19	GSE3467	16365291	[33]	Thyroid Cancer	hsa05216	Thyroid
20	GSE3678		-	Thyroid Cancer	hsa05216	Thyroid
21	GSE9476	17910043	[34]	Acute myeloid leukemia	hsa05221	Blood, Bone marrow
22	GSE18842	20878980	[35]	Non-Small Cell Lung Cancer	hsa05223	Lung
23	GSE19188	20421987	[36]	Non-Small Cell Lung Cancer	hsa05223	Lung
24	GSE3585	17045896	[37]	Dilated cardiomyopathy	hsa05414	Heart

Each data set comes from tissues affected by a specific disease. The KEGG pathway describing that disease is henceforth considered to be the target pathway. The analysis methods were compared in terms of their ability to rank the target pathway as high as possible in the analysis of each data set.

## Methods

### Existing methods

The two methods we chose to compare PADOG against are the *Gene Set Enrichment Analysis* (GSEA) [11] and the *Gene Set Analysis* (GSA) [12]. Briefly, GSEA works as follows. Let *GS*_*i*_ denote the *i*^*th*^ gene set, where *i*=1*..*_*NGS*_. For each gene *j* on the array, GSEA computes a t-statistic *z*_*j*_ for the differential expression of the gene between the disease group and the control group. A gene set score *S*(*GS*_*i*_) is computed similar to a signed version of the Kolmogorov-Smirnov statistic between the values _*zj*_*j*∈*G*_*Si*_and their complement (genes measured on the array but not belonging to the gene set). The class labels of the arrays are permuted and the significance of the gene set score is assessed by determining the null distribution of the gene set score.

The *Gene Set Analysis* (GSA) [12] differs from GSEA in two ways. Firstly, the gene set summary statistic used is the maxmean statistic, defined as:

(1) $$S_{\max}(GS_{i})=\max\left(\sum \overset{\left(+\right)}{\underset{j}{z}}/n,\sum \overset{\left(-\right)}{\underset{j}{z}}/n\right)$$

where the (+) and (-) signs identify the positive and negative *t*-scores respectively, and *n* represents the number of genes in the gene set. Secondly, GSA differs from GSEA by re-standardizing the gene set scores by taking into account scores from sets formed by random selection of genes. Permutations of class labels are then used to infer the significance of the standardized gene set scores. The need for re-standardization is justified by the fact that, given that the genes are correlated (they tend to have either high or low *t*-scores simultaneously), the gene set score computed with the true class labels will be systematically larger than with permuted class labels and, hence, the significance of all gene sets will be overstated.

### Pathway Analysis with Down-weighting of Overlapping Genes (PADOG)

Let *GS*_*i*_ with *i*=1*..*_*NGS*_be the collection of gene sets to be analyzed, each containing *N*(*GS*_*i*_) genes, and *G* be the set of all genes measured on the array that can be mapped to at least one gene set to be analyzed. Then let ${풯}_{g}$ be the value of a moderated *t*-score [15] of the gene *g* between the two conditions of interest with *g*∈*G*. The moderated *t*-scores are similar to ordinary *t*-scores, except that their standard errors have been moderated across genes, i.e., shrunk towards a common value using a Bayesian model [15]. The moderated t-scores are expected to be more reliable than ordinary t-scores because the shrinkage of the gene standard deviations will prevent large t-scores to occur only due to small gene standard deviations.

Moreover, let *f *(*g*) be the frequency of gene *g* across all gene sets to be analyzed. Here *f *(*g*) can take values from 1 to *N*_*GS*_ since a gene can be either specific to a gene set by appearing only in that gene set, or it is present in all gene sets, respectively. We want to weight the *t*-scores of the genes with a function of their frequency in such a way that the most frequently appearing gene gets a weight of w=1.0, while gene set specific genes get double weight (w=2.0). We chose a monotonically decreasing function to relate the gene weight *w*(*g*) to the gene frequency *f *(*g*) so that it is bounded between 1.0 and 2.0 and drops faster with increasing frequency values:

(2) $$w(g)=1+\sqrt{\frac{\max(f)-f(g)}{\max(f)-\min(f)}}$$

**Figure 1:**
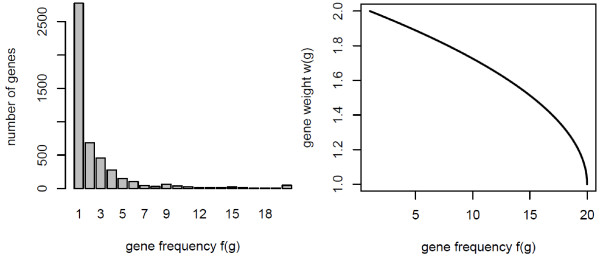
**The weighting function used in PADOG.** The left panel shows the distribution of gene frequencies across the set of KEGG non-metabolic pathways. About 42% of genes that appear in at least one pathway appear also in other pathways. Gene frequencies over the 99^*th*^ percentile of frequencies, i.e. over 20, were replaced with the value 20. The right panel shows the gene weight (Eq. 1) as a function of gene frequency.

For illustration purposes, the distribution of gene frequencies across all 143 KEGG non-metabolic pathways (treated here as gene sets), as well as the dependency of gene weights on gene frequency, is shown in Figure 1. For each gene set we compute a score as:

(3) $$S_{0}(GS_{i})=\frac{1}{N(GS_{i})}\underset{g\in GS_{i}}{\sum }|풯(g)|\cdot w(g)$$

The formula above describes the gene set scores as the mean across all genes in the gene set of the weighted absolute moderated *t*-scores. The gene set scores obtained with the formula above are first standardized using a row randomization approach described in [12] to yield *S’*_0_(*GS*_*i*_). The row randomization consists of subtracting the mean and dividing by the standard deviation of gene set scores that could be obtained by randomly selecting sets of genes with the same size as the current gene set. Given that our gene set summarization function Eq. 2 is essentially a mean (of absolute weighted moderated t-scores) both the row standardization mean and standard deviation can be inferred from the mean and standard deviation of |풯(g)|·w(g) values of all genes on the array, as the central limit theorem would suggest, and hence no actual permutations are needed. More specifically, the row randomization mean for gene set *GS*_*i*_ will given by the mean (of absolute weighted moderated t-scores) of all genes on the array, and the row randomization standard deviation can be calculated as the standard deviation of |풯(g)|·w(g) values of all genes on the array divided by $\sqrt{N(GS_{i})}$. A second standardization is applied by subtracting the mean and dividing to the standard deviation of *S’*_0_(*GS*_*i*_) scores across all *N*_*GS*_ gene sets to obtain the observed standardized scores, $S_{0}^{*}(GS_{i})$. The probability *P*_*PADOG*_(*GS*_*i*_) to observe such a large or larger standardized score is determined by permuting *N*_*ite*_=1000 times the array/samples labels:

(4) $$P_{\mathrm{PADOG}}(GS_{i})=\frac{\underset{\mathrm{ite}}{\sum }I(S_{\mathrm{ite}}^{*}(GS_{i})\geq S_{0}^{*}(GS_{i}))}{N_{\mathrm{ite}}}$$

where *I* is a function that returns 1 when the argument is true and 0 otherwise, and $S_{\mathrm{ite}}^{*}(GS_{i})$ represents the standardized score obtained with the *ite*-th permutation of the samples for gene set *GS*_*i*_.

### Assessing the sensitivity and gene set ranking capability using real data

**Figure 2:**
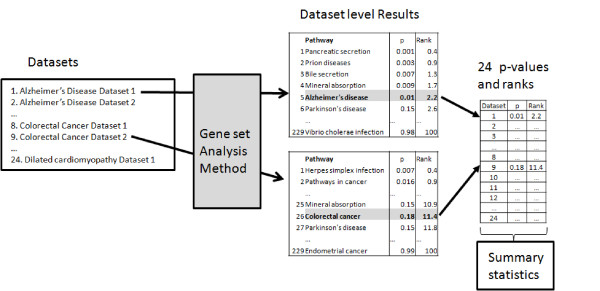
Obtaining ranks and p-values of the target pathways for a given gene set analysis method based on a collection of 24 datasets.

To assess the sensitivity and ranking capability of the gene set analysis methods discussed in this paper, we identified in the Gene Expression Omnibus (GEO) [38], 24 microarray data sets each involving a particular disease. For each such disease, we considered the KEGG pathway that describes the biological phenomena taking place in that disease as the target pathway. For instance, the *Alzheimer’s disease* pathway is the target pathway for all Alzheimer data sets, etc. Table 1 shows the details about these 24 datasets. For most diseases considered, there are several associated data sets in this collection. The gene set analysis methods were compared in terms of their ability to produce low *p*-values, and rank at the top these *target* pathways (one in each data set). A schematic representation of the benchmark system used to assess the performance of each gene set analysis method is shown in Figure 2. There were three categories of statistics computed to compare the performance of the gene set analysis methods considered in this study: 

1. Statistics that describe the distribution of the 24 target pathway’s ***p*-values**, including the geometric mean and median (the lower the better), and the percentage of target pathways with nominal *p*<0.05 (the higher the better). This later statistic is an estimate of the sensitivity of a given analysis method. The percentage of target pathways with False Discovery Rate [39] corrected p-values (called q-values) less than 0.05 is also given.

2. Statistics that describe the distribution of the 24 target pathways **ranks**, including mean and median (the lower the better). The rank of a target pathway, having the ^*ith*^smallest *p*-value amongst all *N*_*GS*_ pathways analyzed for a given dataset, will be equal to *i*/_*NGS*_·100.

3. Statistics that allow to determine if a given pathway analysis method produces better rankings than a reference method, chosen to be GSA since it was the best among the two published methods that we tested. A simple method to test that the ranks produced by a given method for the 24 target pathways are smaller (better) than the reference method would be to use a one-tailed paired Wilcoxon test, the pairing being at data set level. However, the Wilcoxon test assumes that the different ranks are independent between the 24 datasets, yet this is may not be the case because some ranks are obtained for the same pathway in up to 4 datasets (see Table 1). Another approach that we used to analyze the ranks while accounting for the eventual lack of independence among them was to fit a linear mixed-effects model. The dependent variable in this model were the rank values, while the explanatory variables were the analysis method (factor with two levels, with the reference level being GSA) and the dataset ID (to reflect that the ranks are paired at the dataset level), while the random effects were the pathway IDs. Both the coefficient, and one-tailed *p*-value that a given analysis method produces better (smaller) ranks than the reference method were reported.

Note that the gene set analysis methods could have been compared also in analyzing gene ontology terms instead of pathways, however, choosing one GO term most relevant for each dataset would have been more subjective.

### Assessing the sensitivity of gene set analysis using simulated data

A sensitivity comparison between GSEA, GSA and PADOG using simulated data was performed as in [12], but further expanded to also allow for overlap between gene sets. Expression data for 1000 genes and 100 samples (50 in each condition) is generated from a random normal distribution *N*(0,1). A number of 50 gene sets of size 20 were created, with the expression levels of some of the genes in gene set 1 (*GS*_1_) being artificially altered to make only this gene set relevant to the phenotype. Expression levels of genes in *GS*_1_ were changed according to the following 5 scenarios by varying the amount of change, the number of genes that change in the gene set, as well as the proportion of up- to down-regulated genes: 

1. Level of the first 15 genes of *GS*_1_ was increased by 0.3 units in group 2.

2. Level of the first 10 genes of *GS*_1_ was increased by 0.3 units and the level of the next 5 genes was decreased by 0.3 units in group 2.

3. Level of the first 8 genes of *GS*_1_ was increased by 0.3 units and the level of the next 7 genes was decreased by 0.3 units in group 2.

4. Level of the first 7 genes of *GS*_1_ was increased by 0.4 units and the level of the next 3 genes was decreased by 0.4 units in group 2.

5. Level of the first 5 genes of *GS*_1_ was increased by 0.4 units and the level of the next 5 genes was decreased by 0.4 units in group 2.

A number of 50 data sets were generated for each of the six scenarios above. Orthogonal on the different scenarios we considered three analysis setups that could influence the results of PADOG but not GSA and GSEA, according to whether or not the genes in *GS*_1_ are allowed to be present in other gene set as well (e.g. (*GS*_50_)). In the first setup I), *GS*_1_ did not overlap with other gene sets as in [12], II) All genes designed to be DE in *GS*_1_ were included also in *GS*_50_, and III) All non-DE genes of *GS*_1_ were included in *GS*_50_. With setup I) we are basically interested in assessing if the gene set summarization function of PADOG (mean or absolute values) combined with the moderated t-scores compares favorably to GSA and GSEA, because in the absence of overlap, the genes of *GS*_1_ will have the same weight (w=1.0). When the DE genes in *GS*_1_ appear also in other gene sets but the non-DE do not (setup II), PADOG is expected to give higher p-values to *GS*_1_ compared to the situation when there is no overlap. This is because the weight of the DE genes in this case will be lower than the weight on non-DE genes. In contrary, if the genes that are non-DE in *GS*_1_ overlap but the DE genes are specific to *GS*_1_ (setup III) then PADOG is expected to produce smaller p-values for *GS*_1_ because the DE genes will have more weight and also larger t-scores.

### Assessing the specificity of gene set analysis

To test the ability of the gene set analysis methods to not reject the null hypothesis when it is true, i.e. their specificity, we conducted two simulation studies.

#### Simulation of the null hypothesis by sample labels permutation

In the first simulation study all the 24 data sets were considered, but their array/samples class labels were permuted at random before analysis so that the correlation structure between genes is preserved. In 100 different trials, we computed several of the statistics described above, including the median of target pathways *p*-values, median ranks, and the percentage of pathways with *p*<0.05. The average of these statistics over the 100 trials are reported.

The purpose of this simulation was two-fold. First, it allows us to determine if the target pathways-based benchmark works, i.e. if the ranking results are worse for all methods when the labels are permuted compared to when the true class labels are used. Second, it allows us to estimate the false positive rate (1-specificity) of each gene set analysis method and compare it with the level expected under the null hypothesis. All analysis methods were run on *the same* 100 permutations of the original class labels of each of the 24 data sets to eliminate any differences introduced by random chance. The number of internal iterations used by each analysis method was *N*_*ite*_=500.

#### Simulation of the null hypothesis by generating random data

At the suggestion of one of the reviewers, a second type of simulation was performed to determine the false positives rate of gene set analysis methods by generating random data from a normal distribution with mean 0 and standard deviation of 1, N(0,1). For each of the 24 real datasets, 50 fake replicas were created by maintaining the actual sample size and number of genes but generating data at random, for a total of 1200 simulated datasets. The structure of the gene sets was preserved as defined by the 229 KEGG metabolic and non-metabolic pathways, therefore maintaining a meaningful overlap between the different genes in the gene sets. The fraction of all significant pathways (false positive rate) at different *α*thresholds was determined.

### Data Analysis

For all 24 datasets shown in Table 1 which were available from the Gene Expression Omnibus (GEO), the analysis was performed consistently by: a) removing outlier arrays (if necessary), b) log transforming the data and normalizing it, c) performing a moderated t-test between groups and computing probes/probesets *p*-values, d) resolving duplicate probes/probesets to Entrez ID mappings by keeping the probe/probeset with smallest *p*-value for each unique gene and, e) filtering out all genes that could not be mapped on any of the pathways. The normalization of datasets obtained on Affymetrix arrays was performed using the RMA algorithm [40] implemented in the *affy*[41] package of Bioconductor[42], while normalization of of datasets run on Illumina arrays were normalized using the quantile normalization algorithm [43] implemented in the *preprocessCore* of Bioconductor. The package *limma*[44] was used to compute a moderated two-sample paired or unpaired *t*-score depending on the particular design of each experiment.

The GSEA analysis was performed using the R implementation available freely at http://www.broadinstitute.org/gsea/index.jsp, while the GSA analysis was performed using the *GSA* R package [45]. PADOG was implemented in R as well, together with the validation benchmark system comparing the methods. All methods were run using 1,000 iterations to estimate the pathway *p*-values shown in Tables 2,3,4 and 5, while 500 iterations were used in the specificity analysis results shown in Table 6 and 7.

**Table 2:** Comparison between gene set analysis methods in terms of sensitivity and pathway ranking when analyzing 143 KEGG non-metabolic pathways

	**GSEA**	**GSA**	**PADOG**
*p* geometric mean	0.2846	0.1516	**0.0585**
*p* median	0.2468	0.147	**0.1225**
% *p*<0.05	0	12.5	**33.3**
% *q*<0.05	0	0	**4.2**
rank mean	42.31	28.64	**21.45**
rank median	35.84	21.15	**14.69**
Wilcoxon *p*	0.9885	reference	**0.0007**
LME *p*	0.9909	reference	**0.0008**
LME coefficient	13.67	reference	**-7.20**

The table shows statistics computed from nominal and adjusted *p*-values, and ranks of the 24 target pathways *only*, including geometric mean, median and percentages of pathways significant at 0.05 level based on nominal and adjusted p-values (q-values). The results of comparing the ranks of each method against GSA method (chosen as reference), using a paired Wilcoxon test and a linear mixed-effects model, are included. The best value for each criterion is shown in bold.

**Table 3:** Comparison between pathway analysis methods in terms of sensitivity and pathway ranking when analyzing 229 KEGG metabolic and non-metabolic pathways

	**GSEA**	**GSA**	**PADOG**
*p* geometric mean	0.2846	0.1387	**0.0485**
p median	0.2468	0.142	**0.091**
% *p*<0.05	0	16.7	**33.3**
% *q*<0.05	0	0	**4.2**
rank mean	41.42	26.97	**18.95**
rank med	38.43	16.7	**13.05**
Wilcoxon *p*	0.9956	reference	**0.0006**
LME *p*	0.9962	reference	**0.0023**
LME coefficient	14.45	reference	**-8.02**

The table shows statistics computed from nominal and adjusted *p*-values, and ranks of the 24 target pathways *only*, including geometric mean, median and percentages of pathways significant at 0.05 level based on nominal and adjusted p-values (q-values). The results of comparing the ranks of each method against GSA method (chosen as reference), using a paired Wilcoxon test and a linear mixed-effects model, are included. The best value for each criterion is shown in bold.

**Table 4:** A sensitivity analysis using simulated data in the absence and presence of overlap between gene sets

	**Scenario**	**GSA**	**GSEA**	**PADOG Setup I**	**PADOG Setup II**	**PADOG Setup III**
	1	**5e-04**	*0.0015*	0.0121	0.0378	0.0067
	2	0.0276	0.225	*0.0113*	0.0374	**0.0059**
	3	0.0654	0.2539	*0.0133*	0.0397	**0.0111**
	4	0.0103	0.1535	*0.0018*	0.0271	**3e-04**
	5	0.0161	0.2352	*0.0011*	0.016	**1e-04**

The table shows the mean p-values for *GS*_1_ (designed to be relevant to the phenotype) over 50 different trials in each of the 5 different scenarios. GSA and GSEA p-values do not change if genes in *GS*_1_ are found in other gene sets as well. Results for PADOG are given in the absence of overlap (Setup I), presence of overlap between the genes designed to be DE in *GS*_1_ and other gene sets (Setup II), and presence of overlap between the non-DE genes of *GS*_1_ and other gene sets (Setup III). All methods used 1000 permutations to compute the two sided p-values for *GS*_1_. Best values are shown in bold and second best are italicized.

**Table 5:** Determining the contribution of gene weighting and moderated t-scores in PADOG when analyzing 229 KEGG metabolic and non-metabolic pathways

	**noM**	**noW**	**PADOG**	**noMnoW**
p geomean	0.0480	0.1330	**0.0486**	0.1225
p med	0.092	0.1695	**0.091**	0.1595
% p.value<0.05	**33.3**	16.7	**33.3**	16.7
% q.value<0.05	**8.3**	0	4.2	0
rank mean	20.52	22.33	**18.95**	22.48
rank med	14.38	15.71	**13.05**	16.81
p Wilcox.	0.0260	0.371	**0.002**	reference
p LME	0.0463	0.314	**0.0030**	reference
coef. LME	-1.96	-0.15	**-3.53**	reference

The table shows statistics computed from nominal and adjusted *p*-values, and ranks of the 24 target pathways *only*, including geometric mean, median and percentages of pathways significant at 0.05 level based on nominal and adjusted p-values (q-values). The results of comparing the ranks of each method against noMnoW method, using a paired Wilcoxon test and a linear mixed-effects model, are included. The best value for each criterion is shown in bold. PADOG is compared against simpler approaches that i) use gene weights but regular rather than moderated t-scores (noM), ii) use moderated t-scores but no gene weights (noW) and iii) use neither moderated t-scores nor gene weights (noMnoW).

**Table 6:** Comparing gene set analysis methods performance under the null hypothesis simulated by class labels permutation

	**p median**	**Rank median**	**% p.value<0.05**
PADOG	0.49	48.9	4.9
GSA	0.51	50.6	5.3
GSEA	0.50	50.1	5.0

The medians of target pathways *p*-values and ranks, as well as the fraction of target pathways with *p*<0.05 were computed in 100 simulation trials. At each trial the class labels of the samples in each of 24 real datasets were permuted before analysis. The averages statistics over the 100 trials are shown.

**Table 7:** False positive rates when null hypothesis is simulated by generating random expression data

	***α=*0.05**	***α=*0.01**
PADOG	0.051	0.012
GSA	0.052	0.015
GSEA	0.052	0.012

The fraction of all pathways significant at *α*=0.05 and *α*=0.01 were computed after applying the three analysis methods on 1200 datasets having expression data generated from a random normal N(0,1) distribution. The collection of gene sets used in the analysis was defined by the 229 KEGG non-metabolic and metabolic pathways.

The set of 229 metabolic and non-metabolic pathways and their genes were obtained from the *KEGG.db* annotation package [46] of Bioconductor [42]. The split between metabolic and non-metabolic pathways was done based on KEGG’s classification.

All analyses were run under the R statistical language and environment [47] version 2.14 and using other infrastructure packages available in Bioconductor version 2.9.

## Results and discussion

### Sensitivity and rank analysis using real data

We compared the PADOG method proposed here with two existing methods (GSA and GSEA). The analysis was performed on i) 143 non-metabolic pathways (which included all target pathways) and ii) 229 metabolic and non-metabolic KEGG pathways. The criteria used in the comparison between these methods were the sensitivity, the ranking, as well as the specificity of the gene set analysis methods considered. Table 2 shows the summary of gene set analysis results for the three different methods based on the panel of 24 datasets described in Table 1 when analyzing only KEGG non-metabolic pathways.

**Figure 3:**
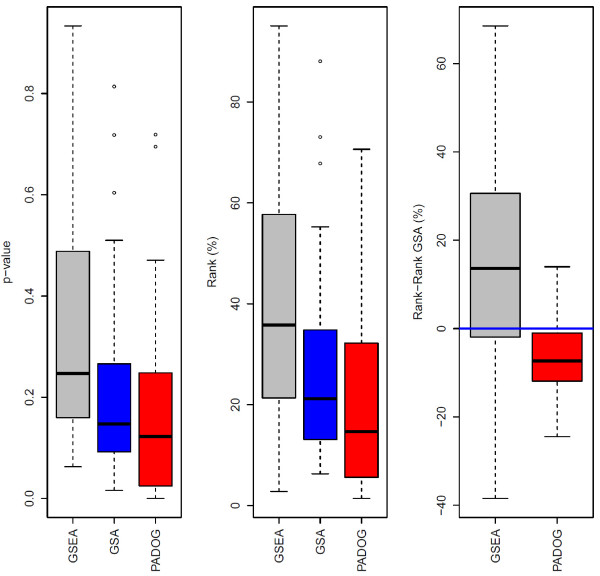
**Comparing the p-values and ranks of target pathways between gene set analysis methods when analyzing 143 KEGG non-metabolic pathways.** The boxplots show the distribution of the target pathways p-value (left panel) and ranks (middle panel), as well as the paired difference in ranks with respect to GSA, chosen as reference method (right panel). The lower the p-values, ranks and ranks differences, the better method.

PADOG compared favorably to both GSA and GSEA in terms of median and geometric mean *p*-values of the target pathways (which are expected to be relevant). Eight (33.3%) of the 24 target pathways were found to be significant (with a *p*-value less than 0.05) with PADOG, but only three did so with GSA (12.5%), and none with GSEA. PADOG was the only method to identify one (4.2%) of the 24 target pathways as significant after adjusting for multiple testing. In terms of the rank that each target pathway received in its data set (sorting pathways by *p*-values), PADOG produced significantly better (lower) rank values compared to GSA, as evaluated by both a paired Wilcoxon test (p=0.0007), and a linear mixed-effects model (p=0.0008). This later test accounts for the fact that the same disease pathway is the target pathway in up to 4 data sets (see Table 1). PADOG improves (reduces) the rank of target pathways by 7.2 rank units compared to GSA, which in turn is better than GSEA by 13.7 units. In other words, on average across the 24 data sets, the target pathways are ranked by PADOG approximately 7 rank units better than GSA, and approximately 21 rank units better than GSEA. The paired difference in ranks for the target pathways between pathway analysis methods and the GSA method, chosen as reference, are also shown using box plots in Figure 3.

**Figure 4:**
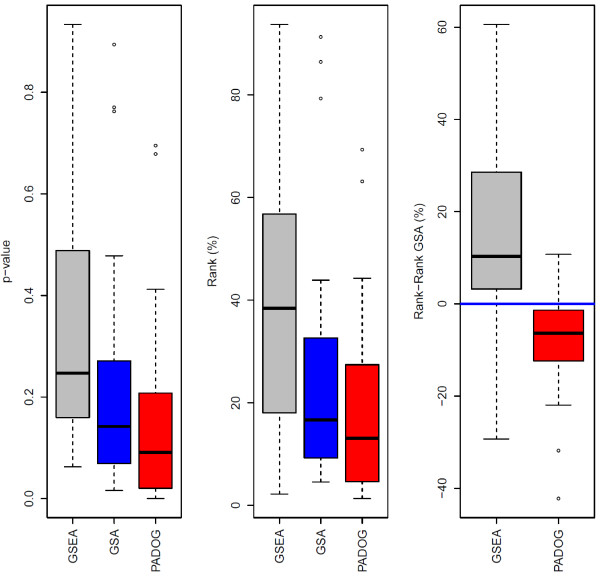
**Comparing the p-values and ranks of the target pathways between gene set analysis methods when analyzing 229 KEGG non-metabolic and metabolic pathways.** The boxplots show the distribution of the target pathways p-value (left panel) and ranks (middle panel), as well as the paired difference in ranks with respect to GSA, chosen as reference method (right panel). The lower the p-values, ranks and ranks differences, the better method.

To determine the robustness of PADOG with respect to changes in the collection of gene sets to be analyzed changes, we have run the same comparison shown in Table 2, on the entire set of 229 KEGG human pathways (metabolic and non-metabolic). An increase in the number of gene sets to be analyzed for a fixed gene expression dataset, is expected to impact the various methods in different ways. With PADOG, when there are more gene sets and, hence, more genes to be analyzed, the moderated *t*-scores of genes in all gene sets are expected to change because the shrinkage of standard deviations in the *t*-scores is based on a larger pool of genes [15]. Secondly, the exact weights assigned to genes in PADOG depend on the number of gene sets in which they appear so these gene weights also change when the collection of gene sets to be analyzed changes. Table 3 and Figure 4 show that PADOG performed favorably compared to the other methods, and that the gains in terms or ranking and sensitivity are robust to changes in the collection of gene sets to be analyzed. Moreover, unlike any other method tested, PADOG identified one (4.2%) of the 24 target pathways as significant after adjusting for multiple testing.

### Sensitivity analysis using simulated data

The result of the sensitivity analysis based on 50 simulated data sets in each of the 5 different scenarios are given in Table 4. These results show that when all genes designed to be differentially expressed (DE) in *GS*_1_ are changing in the same direction (scenario 1), GSA and GSEA have an advantage over PADOG while the opposite is true in all remaining 4 scenarios. These results can be understood by considering the fact that GSA and GSEA statistics are designed to find such cases when all the genes in the gene set change in the same direction while PADOG’s summary statistic is more flexible to accommodate cases when the changes occur in both directions. When the overlap favors the DE genes in *GS*_1_ (Setup III), that is, when its DE genes are specific to this gene set while its non DE-genes are not specific to the gene set, the performance of PADOG increase in all scenarios 1 through 5, as compared to the absence of overlap. However, even when the overlap is not favorable to *GS*_1_ (setup I), that is, when all its non-DE genes are specific to this gene set, PADOG still performes better than GSA and GSEA under scenarios 2 through 5.

### Sources of improvement in PADOG

**Figure 5:**
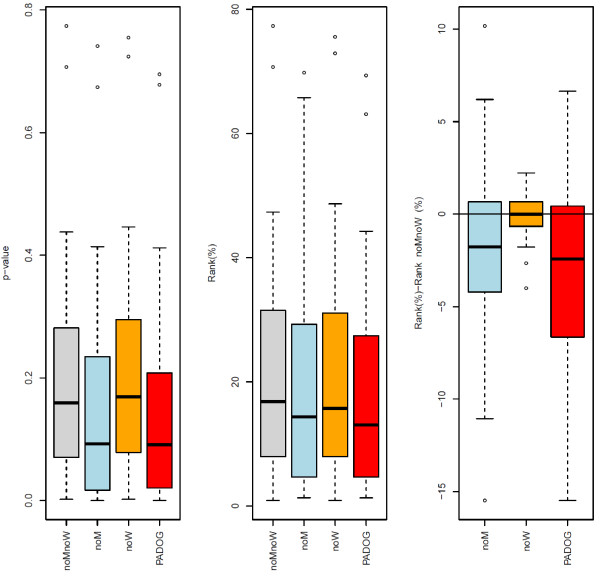
**Determining the contribution of gene weighting and moderated t-scores in PADOG performance.** The boxplots show the distribution of the target pathways p-value (left panel) and ranks (middle panel), as well as the paired difference in ranks with respect to noMnoW, chosen as reference method (right panel). The lower the p-values, ranks and ranks differences, the better method. PADOG is compared against simpler approaches that i) use gene weights but regular rather than moderated t-scores (noM), ii) use moderated t-scores but no gene weights (noW) and iii) use neither moderated t-scores nor gene weights (noMnoW).

The use of gene weights is the main source of improvement with PADOG in terms of ranking and power. This is shown in Figure 5 and Table 5 in which PADOG is compared with simpler alternative methods that i) use gene weights but regular rather than moderated t-scores (*noM*), ii) use moderated t-scores but no gene weights (*noW *) and iii) use neither moderated t-scores nor gene weights (*noMnoW *). As it can be seen in Figure 5 left panel both methods that do not use weights (noW and noMnoW) give higher (worse) p-values for the target pathways than the two other methods that use weights (PADOG and noM). Also as, shown in Table 5, the use of moderated t-scores alone (noW) does not improve the raking compared to the reference (noMnoW) (mean rank is 22.3 vs 22.5 respectively). Although the use of weights (noM) improves the ranking significantly compared to the reference method (noMnoW), the improvement is higher in the presence of the moderated t-scores.

### Specificity analysis of gene set analysis methods

Two simulation studies were performed to determine whether the improved sensitivity of the PADOG method, i.e. producing lower *p*-values for the target pathways, comes at the expense of reduced specificity (increased false positive rate). Table 6 shows three of the same statistics introduced in Table 2 (median *p*-values, median ranks, and percentage of pathways with *p*<0.05) except that their average was taken over 100 trials in which the class labels of the arrays in all 24 datasets were randomly permuted before the analysis. The percentage of target pathways with *p*<0.05 is now the false positive rate (FP) because using random class labels models the null hypothesis in which expression levels are dissociated from the studied phenotypes, yet the gene-gene correlations are preserved. Under these circumstances, any pathways that are reported as significant by any method are, in fact, false positives.

Table 6 shows that, under the null hypothesis, the average median *p*-values, median ranks and fraction of pathways with *p*<0.05 across the 100 random permutations are 0.49, 48.9% and 0.049, respectively for PADOG and similar values are obtained for GSA and GSEA. This is expected since when class labels are permuted, the *p*-values of the target pathways should be uniformly distributed between between 0 and 1 (expected mean 0.5), and rank values should be uniformly distributed between 1/_*NGS*_·100=0.44 and 100 (expected mean 50.22) where *N*_*GS*_ is the number of gene sets analyzed. Table 6 also shows that the average median *p*-values and median ranks are much above (worse) than the level they had when true class labels were used in the analysis (see Table 2). This is the case for all analysis methods. These results prove that: i) the target pathways were indeed in average relevant to their respective phenotypes, ii) the benchmark system was sound, and iii) both the novel, as well as the existing methods were correctly deployed.

An additional simulation in which 1200 datasets were generated by drawing random values from a normal distribution has yielded similar results as the previous simulation. In this case the false positives rate was estimated as the fraction of all pathways across all 1200 datasets with a p-value less than a given threshold *α*. The estimated false positive rates of all three methods were very close to the expected *α* levels as shown in Table 7. This again confirms that PADOG is not expected to find significant gene sets more often than expected by chance regardless if gene are correlated (as in the simulation above) or not (this simulation).

### Specificity analysis of the set of target pathways

In response to the suggestion of one of the reviewers, we aimed at determining how specific the target pathways were to their respective conditions. Given that the phenotype in 16 out of the 24 datasets used in our sensitivity assessment benchmark study is a form of cancer, we determined if the target pathway for each of these cancer types, in average, is found to be more significant than other general pathways typically associated with cancer such as *Apoptosis*, *Cell cycle*, *Pathways in cancer*, and *RNA polymerase*. Table 8 shows that in average on the 16 cancer datasets PADOG shows the strongest evidence (smallest p-values and rank statistics) for association between the pehnotype of the dataset and KEGG’s disease specific pathway for the phenotype (target pathway). The target pathway was preferred by all three methods to any other generic cancer related pathway that we have included in this comparison, based on median p-values and, by PADOG and GSA based on median ranks as well. The *Pathways in cancer* gene set came in a close second for both PADOG and GSA. While for *Apoptosis* and *Cell cycle* the median p-values and ranks were around 25% for all methods, for the *RNA polymerase* pathways these values were above 0.5. This analysis provides evidence that the target pathways we chose were indeed specific for their respective phenotypes.

**Table 8:** A specificity analysis of the target pathways on 16 cancer data sets

**Pathway type**	**Statistic**	**GSEA**	**GSA**	**PADOG**
Target	p med	**0.2603**	**0.087**	**0.043**
Target	rank med	39.56	**10.15**	**6.42**
Apoptosis	p med	0.3329	0.203	0.1985
Apoptosis	rank med	*37.56*	24.24	28.76
Cell cycle	p med	*0.3133*	0.325	0.227
Cell cycle	rank med	**26.29**	36.35	28.61
Pathways in cancer	p med	0.351	*0.114*	*0.0465*
Pathways in cancer	rank med	47.54	*13.21*	*8.41*
RNA polymerase	p med	0.5	0.681	0.6485
RNA polymerase	rank med	57.78	71.51	63.33

The table shows a comparison between the pathways specifically designed by KEGG for each type of cancer (Target pathways) and other pathways that are commonly involved in many cancers. The table shows statistics computed from nominal *p*-values, and ranks of each type of pathway for the 16 cancer datasets shown in Table 1. PADOG gives the most significant p-values and best ranks to the target pathways. For each analysis method, the values for type of pathway with the smallest median p-values and ranks (strongest association with the phenotype) are shown in bold, while the second smallest values are italicized.

## Conclusions

The original contribution of this paper is two-fold. Firstly, this paper introduces the idea of gene weighting in gene set analysis on the basis of gene frequency across the gene sets to analyzed. The reasoning behind this type of gene weighting is that whenever a gene belongs to multiple gene sets, that particular gene is less useful in prioritizing among those gene sets. Conversely, the differential expression of a gene that is present only on a single gene set/pathway represents a stronger evidence that the given gene set/pathway is impacted in the given condition. A second original contribution is the validation procedure deployed here. The classical approach involves analyzing a handful of selected data sets and discussing the results in the light of the existing literature. This is subjective and makes the comparison of various methods practically impossible. The validation proposed here involves the analysis of a large number of data sets (24 in this case) that can be objectively associated with a target gene set/pathway. This objective association is based on the fact that the samples analyzed are collected from tissues affected by the target disease (e.g. in the analysis of colorectal cancer samples, the colorectal cancer pathway is chosen as the target pathway, etc.). This approach allows a comparison of analysis methods in terms of sensitivity and ranking. Such a comparison is: a) objective, b) reproducible, and c) independent of the accuracy and thoroughness of a literature search. Using this approach, we have shown that PADOG is able to identify the target pathways as significant more frequently and rank them consistently higher than two of the best existing methods for the analysis of gene sets based on high-throughput gene expression data.

## Competing interests

The authors of the manuscript do not hold or intend to apply for a patent. However, one or more authors may file an employee invention disclosure form to notify their employer that the work leading to the manuscript may be patentable.

## Author’s contributions

ALT designed and implemented the research and drafted the manuscript. ALT, SD, and RR evaluated the research and improved the manuscript. GB collected the datasets and performed data pre-processing. All authors read and approved the final manuscript.
